# MicroRNAs expression profiling of eutopic proliferative endometrium in women with ovarian endometriosis

**DOI:** 10.1186/1477-7827-11-78

**Published:** 2013-08-15

**Authors:** Piotr Laudanski, Radoslaw Charkiewicz, Mariusz Kuzmicki, Jacek Szamatowicz, Alicja Charkiewicz, Jacek Niklinski

**Affiliations:** 1 Department of Perinatology, Medical University of Bialystok, ul. Marii Sklodowskiej-Curie 24a, 15-276, Bialystok, Poland; 2 Department of Clinical Molecular Biology, Medical University of Bialystok, ul. Waszyngtona 13, 15-276, Bialystok, Poland; 3 Department of Gynecology and Gynecological Oncology, Medical University of Bialystok, ul. Marii Sklodowskiej-Curie 24a, 15-276, Bialystok, Poland; 4 Department of Pathology, Medical University of Bialystok, ul. Waszyngtona 13, 15-276, Bialystok, Poland

**Keywords:** Endometriosis, Eutopic endometrium, MiRNAs

## Abstract

**Background:**

The eutopic endometrium of women with endometriosis, compared with disease-free individuals, contains certain molecular alterations, including the differential expression of microRNA (miRNA). The aim of the study was to compare the expression of the most relevant miRNAs in the eutopic endometrium of women with and without ovarian endometriosis.

**Methods:**

A total of 46 regularly menstruating patients, 21 patients with ovarian endometriosis and 25 controls, underwent surgery in the proliferative phase of the cycle. The eutopic endometrium was collected through aspirating biopsy prior to laparoscopy. Only patients with advanced (stage III and IV) histopathologically confirmed ovarian endometriosis were included. TaqMan MicroRNA Array Cards were applied to examine the expression of 667 human miRNAs in 10 patients with endometriosis and 10 controls. Custom-made, low-density real-time PCR arrays were used to confirm the expression of 15 selected molecules in 21 endometriosis patients and 25 disease-free individuals.

**Results:**

Of 667 miRNAs, 2 were highly likely to be upregulated and 13 were downregulated in the eutopic endometrium of patients with endometriosis compared with the controls. Validation using real-time PCR showed that hsa-miR-483-5p (p = 0.012) and hsa-miR-629* (p = 0.02) are significantly downregulated in patients with endometriosis.

**Conclusions:**

Changes in the expression of select miRNAs might lead to or be a consequence of an early defect in the physiological activity of the proliferative endometrium, ultimately resulting in the overgrowth of this tissue outside the uterus.

## Background

Endometriosis is defined as the presence of endometrial glands and stroma outside the uterine cavity. It represents a widespread condition with a relevant impact on women’s health. The pathogenesis of endometriosis remains unclear. More than 80 years ago, it was hypothesised that endometriosis might arise from an ectopic implant of endometrial cells in the peritoneum of the pelvic cavity after retrograde menstruation [1]. This theory is, however, questionable because the backward passage of endometrial cells along the fallopian tubes is a physiological process that occurs in most women during normal menstruation [2]. Thus, it has subsequently been suggested that the ectopic implant of endometrial cells after passage through the fallopian tubes might also be facilitated through a disturbed immunological status, reflected through the altered status of different biologically active substances, such as chemokines, matrix metalloproteinases, tumour suppressor genes, microRNAs (miRNAs) and potentially many others [3-6].

MicroRNAs are a recently recognised class of highly conserved, noncoding short RNA molecules (approximately 22 nucleotides) that regulate gene expression at the post-transcriptional level. miRNAs suppress protein synthesis through the inhibition of protein translation from mRNA or the promotion of mRNA degradation, thereby silencing gene expression [7]. A rapidly emerging understanding of the complicated regulatory function of miRNAs in cell biology has recently provided new and interesting clues into the function of gene regulation [8].

Abnormal miRNA expression has been associated with a number of benign gynaecological conditions and different malignancies and fertility disorders [9,10]. Although the miRNA expression profiles in the eutopic and ectopic endometrium from women with endometriosis have previously been revealed in a few independent studies [9,11], and it has been shown that these differences exist between the eutopic secretory endometrium of patients with and without endometriosis [12,13], the expression profile has still not been clearly presented for the proliferative eutopic endometrium. Because the endometrium can be easily and non-invasively collected, this tissue can be used as a potential source for markers of endometriosis, where miRNAs could potentially serve this purpose.

The aim of the present study was to perform miRNA gene expression profiling in the proliferative eutopic endometrium of patients with advanced ovarian endometriosis.

## Methods

The study protocol was approved by the Local Ethical Committee of Medical University of Bialystok, Poland, and signed informed consent was obtained from each patient.

A total of 46 patients were recruited in this study. Endometrial samples were initially obtained from 51 regularly menstruating women, aged 20–35 years, undergoing diagnostic or surgical laparoscopic surgery for non-malignant ovarian lesions. After total RNA isolation and quality control (see *RNA isolation and quality control*), we selected 46 samples for further molecular analysis. Patients with endometriosis (Group I, n = 21) stages III to IV were diagnosed through laparoscopic findings according to the revised American Fertility Society classification of endometriosis [14], and each case was confirmed through histopathology. As a control (Group II, n = 25), we used endometrial tissue from 16 women who underwent surgery as part of an infertility work-up and 9 women who underwent surgery to remove simple ovarian cysts; all controls were without any visible endometriosis during laparoscopy.

Patients with autoimmune disease, pelvic inflammatory disease, adenomyosis, dysfunctional uterine bleeding and those taking non-steroidal anti-inflammatory drugs, GnRH agonists and steroids for the past 3 months were excluded. Endometrial samples were collected through Pipelle suction in the operating room before the laparoscopic procedure, and endometriosis samples were collected during laparoscopy. The endometrial tissue samples were classified through histological dating according to the method of Noyes et al. [15], and only patients in the proliferative phase (from the 6^th^ through the 13^th^ day) of the cycle were included in the study.

### RNA isolation and quality control

A piece of each collected tissue was divided into two halves and placed separately in buffered formaline for histopathological studies and RNAlater solution (Sigma Aldrich, Poland) for molecular analysis. The latter was stored for 24 hours at +4°C, and subsequently the tissues were transferred and stored at −80°C. Total RNA was extracted using the *mir*Vana™ miRNA Isolation Kit (Ambion, Life Technologies, Poland). The RNA quality was assessed using an Agilent Bioanalyzer 2100 and Agilent RNA 6000 Nano kit (Agilent Technologies, Perlan, Poland), and the samples selected for further analysis showed minimum degradation, with an RNA integrity number (RIN) above 9 for all samples. The five excluded samples had RINs between 4 and 6. The RNA concentrations were measured using a NanoDrop 2000c (Thermo Scientific, Biotech, Poland). We also used small RNA microfluidic chips (Agilent Small RNA kit, Agilent Technologies, Perlan, Poland), and only the visual evaluation of an apparent band on the capillary gel of electropherograms was performed, as no algorithm has yet been created for this purpose.

### TaqMan MicroRNA Array Cards

The TaqMan low-density Arrays A and B (TaqMan® Array Human MicroRNA A v2.1 + B v2.0 Cards Set, Applied Biosystems, Life Technologies, Poland), which facilitate the comprehensive coverage of Sanger miRBase v14, were configured in a 384-well format microRNA assays spotted onto a microfluidic card, and these analyses were performed on 10 samples of the eutopic endometrium of patients with ovarian endometriosis and 10 controls. The Micro Fluidic Card contains eight sample-loading ports, each connected by a micro channel to 48 miniature reaction chambers for a total of one sample per card. The details are available at: http://tools.invitrogen.com/content/sfs/manuals/cms_054742.pdf.

Single-stranded cDNA was synthesised from 500 ng of total RNA using the TaqMan® MicroRNA Reverse Transcription Kit (Applied Biosystems, Life Technologies, Poland) and highly multiplexed RNA-specific stem-looped Megaplex RT Primers, Human Pool A v2.1 and Human Pool B v2.0 (Applied Biosystems, Life Technologies, Poland), according to the manufacturer’s protocol.

A 10-μl sample of each cDNA was added to 45 μl of nuclease-free water and an equal volume of 2 × TaqMan Universal PCR Master Mix containing uracil-DNA glycosylase (UNG) (Applied Biosystems, Life Technologies, Poland), and after brief mixing and centrifugation, the mixture was transferred into one of the eight loading ports on a micro fluidic card. The cards were centrifuged in a Sorvall Legend™ centrifuge (Kendro Scientific, Asheville, USA) for 2 min at 1200 rpm (331 × *g*) to distribute the samples from the loading port into each well. Following centrifugation, the cards were sealed with a TaqMan low-density array sealer (Applied Biosystems, Life Technologies, Poland) to prevent cross-contamination. Each card was placed in the micro fluidic card sample block of an ABI Prism® 7900HT sequence detection system (Applied Biosystems, Life Technologies, Poland), and PCR amplification was performed. Thermal cycling conditions were 2 min at 50°C to activate uracil-DNA glycosylase, 10 min at 94.5°C (activation), 40 cycles of denaturation at 97°C for 30 s, and annealing and extension at 59.7°C for 1 min. The raw Ct values were calculated using RQ manager software v1.2.1 (ABI).

### Custom-made TaqMan Low-Density Arrays

The TaqMan Low-Density Array is a custom-designed 16-microRNA-expression assay (Applied Biosystems, Life Technologies, Poland), preconfigured in a 384-well format and spotted onto a microfluidic card (3 replicates per assay). The Micro Fluidic Card contains eight sample-loading ports connected through a micro channel to 48 miniature reaction chambers (16×3) for a total of eight samples per card.

The miRBase IDs (miRBase Accession Number) included in the assay were has-miR-24 (MIMAT0000080), has-miR-885-5p (MIMAT0004947), has-miR-26b (MIMAT0000083), has-let-7b (MIMAT0000063), has-miR-185 (MIMAT0000455), has-miR-142-3p (MIMAT0000434), has-miR-29b (MIMAT0000100), has-miR-483-5p (MIMAT0004761), has-miR-144* (MIMAT0004600), has-miR-145* (MIMAT0004601), has-miR-629* (MIMAT0003298), has-miR-222* (MIMAT0004569), has-miR-497 (MIMAT0002820), has-miR-675 (MIMAT0004284) and has-miR-106b* (MIMAT0004672). The expression of the target microRNA was normalised to the expression of RNU6BsnRNA (miRBase Accession Number NR_004394), as an endogenous control.

In the first step, we prepared the RT primer pool, comprising 16 individual RT primers (Applied Biosystems, Life Technologies, Poland). Subsequently, we used the RT primer pool and the TaqMan® MicroRNA Reverse Transcription Kit (Applied Biosystems, Life Technologies, Poland) to synthesise single-stranded cDNA from total RNA samples according to the manufacturer’s protocols.

PCR amplification and signal detection was performed using the ABI Prism® 7900HT sequence detection system (Applied Biosystems, Life Technologies, Poland) as previously described. The raw Ct values were calculated using the RQ manager software v1.2.1 (ABI) with automatic baseline and threshold settings, according to the manufacturer’s instructions.

### Statistical analysis

#### Preprocessing of TaqMan® Array MicroRNA Cards (Applied Biosystems)

MicroRNA expression was evaluated using TaqMan® Array MicroRNA Cards (A and B) (Applied Biosystems). The cards contain primer sets for 736 human miRNA sequences (667 unique miRNAs), two sequences from *Arabidopsis*, eight measurements of MammU6, and 22 RNU sequences.

In this study, the data were normalised to the endogenous control (EC) MammU6 (RNU6B - small nuclear RNA) [16] using SDS v 2.3 and RQ manager software v1.2.1 (Life Technologies) with automatic baseline and threshold settings.

The expression data were provided as Ct, ΔCt, ΔΔCt, and 2^-ΔΔCt^ values. The ΔCt values were calculated relative to MammU6. The ΔΔCt values were calculated for different reference samples on cards A and B with positive signals.

The ΔCt values were used to generate flag values for the different miRNAs. A flag value is the number of samples fulfilling the following criteria:

*values*: number of samples in which the indicated miRNA was detected

*nd*: number of samples in which the indicated miRNA was not detected (no expression value reported)

*good*: number of samples in which the indicated miRNA reached ΔCt values among the lowest 25% (i.e., miRNAs with strong expression)

*normal*: number of samples in which the indicated miRNA reached ΔCt values between the lowest and the highest 25%

*low*: number of samples in which the indicated miRNA yielded ΔCt values among the highest 25% (i.e., miRNAs with weak expression)

The average values, standard deviations, and coefficients of variance were calculated for the ΔΔCt values of each miRNA in the groups of samples derived from donors with and without endometriosis. Only miRNAs with at least two detectable samples per group were retained for subsequent statistical analysis.

### MiRNAs with differential expression between endometriosis and control samples

Candidate miRNAs with differential expression between the donor groups with endometriosis compared with the donors without endometriosis were identified using a combination of selection methods. First, the p-value was calculated using t-tests with Welsh approximation for unequal variance. In addition, the average and median fold-changes between both sample groups were calculated.

Because high intra-group variability of miRNA expression was observed, a miRNA was considered differentially expressed between the two groups when at least one of the following prerequisites applied:

p-value of 0.1 or better

mean fold-change of 2 or more (−2 or less for downregulation)

median fold-change of 2 or more (−2 or less for downregulation)

The Mann–Whitney-Wilcoxon test was used to compare two groups for quantitative data due to the non-normal distribution of the tested variables. The significance level was equal to 0.05. The calculations were performed using Microsoft Excel and the data analysis software system STATISTICA, StatSoft, Inc. Ver. 7.1.

## Results

The patient characteristics are shown in Table 1. Among the 667 miRNAs on the two cards, 159 molecules passed the selection. The miRNAs identified as potentially differentially expressed were further classified into the three confidence categories “top”, “medium” and “low”.

**Table 1:** Clinical characteristics of the patients

	**Endometriosis (n = 21)**	**Control (n = 25)**
*Age (years)*	31.35 ± 0.90	30.73 ± 0.91
***Infertility, n (%)***	9 (50)	16 (64)
*Primary*	7	8
*Secondary*	2	8
*Duration of infertility (months)*	48.2 ± 19.4 (23–71)	50.8 ± 15.8 (13–71)
***Ovarian cysts, n (%)***		
*Endometrial*	21 (100)	-
*Simple*	-	9 (36)

Data are presented as the means ± SEM. Ranges are provided for “duration of infertility”.

This selection method is an arbitrary method, frequently used for in-house analyses (performed at Miltenyi Biotec) on other microarray projects, where the identification through statistical comparison is complicated due to high variability between replicate experiments.

Specifically it is as follows:

*top*: no single measurement in neither sample group was counted “low”; at least one measurement in both sample groups was counted “good” (15 miRNAs)

*medium*: no single measurement in neither sample group was counted “low”; no measurement in neither sample groups was counted “good” (69 miRNAs)

*low*: at least one measurement in any sample was counted “low” (75 miRNAs)

Interestingly, many of the miRNAs classified in the “low” confidence group were associated with high intra-group ratios (i.e., have large intra-group variability).

Among the fifteen miRNAs in the top group, two miRNAs were highly likely to be upregulated, i.e., hsa-miR-24and hsa-miR-885-5p, whereas thirteen miRNAs were highly likely to be downregulated, i.e., hsa-miR-26b, hsa-let-7b, hsa-miR-185, hsa-miR-142-3p, hsa-miR-29b, hsa-miR-483-5p, hsa-miR-144*, hsa-miR-145*, hsa-miR-629*, hsa-miR-222*, hsa-miR-497, hsa-miR-675 and hsa-miR-106b*, in the eutopic endometrium of patients with endometriosis compared with the controls (Table 2).

**Table 2:** The 15 top-regulated miRNAs, showing high probability of differential regulation between the eutopic endometrium of patients with and without ovarian endometriosis

**Nr**	**miRNA**	**Endometriosis**				**Control**							
		**Mean**	**std. deviation**	**Coefficient of variation**	**Max/min R**	**Mean**	**std. deviation**	**Coefficient of variation**	**Max/min R**	**Mean difference**	**Median difference**	**Mean fold- change**	**Median fold- change**
1	hsa-miR-24	1.29	0.04	3.12	1.089	1.16	0.08	6.87	1.21	1.11	1.17	1.11	1.17
2	hsa-miR-885-5p	4.18	3.92	93.75	12.25	2.06	1.23	59.96	9.22	2.02	1.21	2.02	1.21
3	hsa-miR-26b	1.38	0.43	31.37	2.69	3.04	3.60	118.48	12.84	0.45	0.92	−2.19	−1.08
4	hsa-let-7b	1.002	0.32	32.48	3.11	1.37	0.39	28.64	2.352	0.730	0.870	−1.369	−1.149
5	hsa-miR-185	0.98	0.42	42.89	5.69	1.52	0.74	48.87	4.3	0.69	0.81	−1.54	−1.23
6	hsa-miR-142-3p	1.81	1.07	58.93	8.57	3.03	1.61	53.12	5.43	0.6	0.56	−1.66	−1.76
7	hsa-miR-29b	0.96	0.64	66.84	9.34	1.73	0.96	55.89	5.78	0.55	0.59	−1.79	−1.68
8	hsa-miR-483-5p	0.75	0.58	76.80	22.55	1.34	0.76	56.75	8.29	0.56	0.43	−1.77	−2.31
9	hsa-miR-144*	0.14	0.085	57.82	8.9	0.49	0.29	60.68	6.65	0.3	0.34	−3.31	−2.89
10	hsa-miR-145*	1.04	0.8	77.46	12.37	2.39	1.8	75.38	8.9	0.43	0.34	−2.3	−2.94
11	hsa-miR-629*	1.53	1.352	87.88	15.1	1.9	1.31	69.05	10	0.80	0.48	−1.24	−2.06
12	hsa-miR-222*	0.71	0.44	61.54	16.70	1.47	1.23	83.64	13.48	0.48	0.69	−2.056	−1.44
13	hsa-miR-497	0.61	0.37	60.51	16.68	1.29	1.26	97.74	16.98	0.47	0.58	−2.12	−1.7
14	hsa-miR-675	1.09	0.77	70.73	17.08	2.66	2.26	84.87	12.03	0.41	0.54	−2.42	−1.84
15	hsa-miR-106b*	0.572	0.571	99.96	72.356	1.44	1.9	131.84	39.01	0.39	0.48	−2.53	−2.04

R represents the ratio between the max and min values within one replicate group (see Material and Methods for a detailed description).

Candidate miRNAs in the “top” group were validated using an alternative method and additional samples. A custom-made TaqMan Low-Density Real-Time PCR Array expression analysis was performed for 15 miRNAs on an enlarged group of patients, namely the 21 samples from patients with ovarian endometriosis (stages III-IV) and 23 controls with eutopic endometria.

The results showed that among the 15 miRNAs classified in the top confidence category in the TaqMan Array MicroRNA Cards assays, two miRNAs were significantly different between the studied groups, as measured using the custom-made TaqMan Low-Density Real-Time PCR Array (Table 3). Specifically, hsa-miR-483-5p (p = 0.012) and hsa-miR-629* (p = 0.02) were downregulated, as shown after the initial analysis of cards A and B in patients with endometriosis, showing 1.39 (0.19-7.6) and 1.58 (0.44-3.35), respectively, for the two miRNAs compared with the controls, showing 2.4 (0.15-16.2) and 2.02 (1.4-52), respectively.

**Table 3:** Median relative quantity (RQ) values for the15 top-regulated miRNAs in the eutopic endometrium of patients with and without ova1rian endometriosis

**miRNA**	**Endometriosis median RQ (min-max)**	**Control median RQ (min-max)**	p-value**
hsa-miR-24	0.53 (0.03-1.4)	0.6 (0.002-3.8)	0.58
hsa-miR-885-5p	0.23 (0.06-3.7)	0.23 (0.02-1.3)	0.96
hsa-miR-26b	0.53 (0.02-2.6)	0.65 (0.001-8.3)	0.41
hsa-let-7b	0.24 (0.002-2.2)	0.41 (0.005-4.06)	0.39
hsa-miR-185	0.35 (0.06-1.4)	0.43 (0.05-5.8)	0.32
hsa-miR-142-3p	0.25 (0.003-1.6)	0.29 (0.01-4.3)	0.3
hsa-miR-29b	0.38 (0.08-1.9)	0.22 (0.07-9.4)	0.9
hsa-miR-483-5p	1.39 (0.19-7.6)	2.4 (0.15-16.2)	0.012
hsa-miR-144*	0.24 (0.09-2.4)	0.6 (0.01-19.8)	0.24
hsa-miR-145*	0.98 (0.07-2.6)	1.13 (0.13-23,7)	0.51
hsa-miR-629*	1.58 (0.44-3.35)	2.02 (1.4-52.8)	0.02
hsa-miR-222*	0.36 (0.04-1.8)	0.53 (0.02-6.6)	0.32
hsa-miR-497	0.35 (0.06-2.03)	0.31 (0.04-4.8)	0.74
hsa-mir-675	0.51 (0.001-8.86)	0.94 (0.09-3.8)	0.1
hsa-mir-106b*	0.55 (0.001-2.57)	0.55 (0.002 -2.59)	0.8

** statistically significant value of less than 0.05.

## Discussion

In the present study, we demonstrated that two miRNAs, i.e., hsa-miR-483-5p and hsa-miR-629*, showed lower expression in the eutopic endometrium of women with advanced ovarian endometriosis compared with control subjects.

Recently, miR-483-5p, a conserved sequence encoded by intron 2 of IGF2 (insulin growth factor), was identified [17]. The expression of this miRNA has been characterised in different tissues [18,19], but there are few reports concerning miR-483-5p function. It was previously revealed that miR-483-5p could be coexpressed with its host gene, therefore playing a biological role as a functional partner of IGF2 [20].

It has been demonstrated that IGF2 promoted angiogenesis through the IGF2 receptor (IGF2R) system in vascular endothelial cells, and miR-483-5p overexpression in endothelial cells blocked angiogenesis, while the inhibition of miR-483-5p increased this process [21]. Recently, it was shown that miR-483-5p was significantly downregulated in gliomas. The overexpression of miR-483-5p suppressed glioma cell proliferation and induced a G0/G1 arrest, whereas miR-483-5p inhibition promoted cell proliferation, suggesting that miR-483-5p serves as a tumour suppressor [22]. Because the eutopic endometrium of patients with endometriosis shows features of increased angiogenesis [23], it cannot be excluded that the inhibition of miR-483-5p might lead to the subsequent augmentation of new vessel formation in the ectopic lesion.

Little is known about miR-629*, and the expression of this miRNA has not been measured in previous multi-type cancer datasets [24,25]. Using microarrays to profile more than 700 miRNAs in 28 matched tumour/normal samples from 8 different tumour types (breast, colon, liver, lung, lymphoma, ovary, prostate and testis), it has been shown that miR-629* is overexpressed in all 8 cancer types. Mitchell et al. [26] compared miRNA serum levels between 12 mice with human prostate cancer xenografts and 12 controls. These authors showed that miR-629* is greatly over-expressed in the xenograft mice plasma and proposed that miR-629* is potentially secreted from the xenograft cells. More recently it was shown that miR-629* is actively involved in the microRNA inflammatory feedback loop circuit comprising miR-124, IL6R (interleukin 6 receptor), STAT3 (signal transducer and activator of transcription 3), miR-24 and HNF4a (hepatocyte nuclear factor 4a). The activation of this circuit maintains the suppression of HNF4a and sustains oncogenesis [27]. Although a majority of the components of the above pathway have not been studied in endometriosis so far, it is feasible that the downregulation of miR629* might also be involved in the overexpression of potential inflammatory-related growth-promoting factors.

To the best of our knowledge the current study is the first to compare the miRNA expression profile in the proliferative eutopic endometrium of patients with and without endometriosis. Previous studies have focused on the evaluation of the miRNA expression levels in secretory eutopic endometrial versus ectopic lesions. It has been previously demonstrated that 48 miRNAs are differentially expressed in the paired eutopic and ectopic endometrium and isolated endometrial cells [11]. More recently, microarray analysis has revealed 14 upregulated and eight downregulated miRNAs in paired ectopic and eutopic endometrial tissues, and the obtained data and results of the functional analysis suggest that the 22 miRNAs and their cognate mRNA target sequences constitute pathways, including c-Jun, CREB-binding protein, protein kinase B (Akt), and cyclin D1 (CCND1) signalling, which promotes endometriosis [28].

The obtained results also suggest that lower levels of hsa-miR-483-5p and hsa-miR-629* expression in the proliferative eutopic endometrium contribute to or result from the elevated biological activity of advanced disease. It is feasible that the changes in the expression of these genes might lead to or be a consequence of an early defect in the physiological activity of the proliferative endometrium, ultimately resulting in the overgrowth of this tissue outside the uterus.

A limitation of the present study is that the control group only included infertile women and women with simple ovarian cysts. Theoretically, there are several causes of infertility, and ovarian cysts could influence the expression of genes in the endometrium. In Poland, it is impossible to collect suitable healthy control samples similar to those collected during laparoscopic sterilisation, as sterilisation is considered an illegal form of contraception.

## Conclusions

In the present study, we investigated the expression profile of miRNAs and observed that two miRNAs were significantly downregulated in the eutopic endometrium of patients with endometriosis. Thus, these results provide the foundation for additional functional studies to elucidate the potential role of miRNAs in endometriosis.

## Competing interests

The authors declare that they have no competing interests.

## Authors' contributions

PL conceived and designed the study, performed laboratory work, analysed the data and wrote the manuscript. RC performed molecular biology experiments, particularly the RNA isolation and PCR array experiments. MK assisted in the interpretation and analysis of the data and performed the PCR array experiments. JS assisted in the collection of samples and the interpretation and analysis of the data. AC performed molecular biology experiments, particularly the RNA isolation and PCR array experiments. JN coordinated the molecular biology experiments and analysed the data. All authors read and approved the final manuscript.

## References

[B1] Sampson JA Heterotopic or misplaced endometrial tissue Am J Obstet Gynecol 1925 10 649 664

[B2] Liu DT Hitchcock A Endometriosis: its association with retrograde menstruation, dysmenorrhoea and tubal pathology Br J Obstet Gynaecol 1986 93 859 862 10.1111/j.1471-0528.1986.tb07995.x 3741813

[B3] Harada T Iwabe T Terakawa N Role of cytokines in endometriosis Fertil Steril 2001 76 1 10 10.1016/S0015-0282(01)01816-7 11438312

[B4] Laudanski P Szamatowicz J Kowalczuk O Kuzmicki M Grabowicz M Chyczewski L Expression of selected tumor suppressor and oncogenes in endometrium of women with endometriosis Hum Reprod 2009 24 1880 1890 10.1093/humrep/dep175 19429661

[B5] Laudanski P Szamatowicz J Ramel P Matrix metalloproteinase-13 and membrane type-1 matrix metalloproteinase in peritoneal fluid of women with endometriosis Gynecol Endocrinol 2005 21 106 110 10.1080/09513590500154043 16109597

[B6] Szamatowicz J Laudanski P Tomaszewska I Szamatowicz M Chemokine growth-regulated-alpha: a possible role in the pathogenesis of endometriosis Gynecol Endocrinol 2002 16 137 141 12012624

[B7] Bartel DP MicroRNAs: genomics, biogenesis, mechanism, and function Cell 2004 116 281 297 10.1016/S0092-8674(04)00045-5 14744438

[B8] Fabian MR Sonenberg N The mechanics of miRNA-mediated gene silencing: a look under the hood of miRISC Nat Struct Mol Biol 2012 19 586 593 10.1038/nsmb.2296 22664986

[B9] Olson P Lu J Zhang H Shai A Chun MG Wang Y Libutti SK Nakakura EK Golub TR Hanahan D MicroRNA dynamics in the stages of tumorigenesis correlate with hallmark capabilities of cancer Genes Dev 2009 23 2152 2165 10.1101/gad.1820109 19759263 PMC2751988

[B10] Teague EM Print CG Hull ML The role of microRNAs in endometriosis and associated reproductive conditions Hum Reprod Update 2010 16 142 165 10.1093/humupd/dmp034 19773286

[B11] Pan Q Luo X Toloubeydokhti T Chegini N The expression profile of micro-RNA in endometrium and endometriosis and the influence of ovarian steroids on their expression Mol Hum Reprod 2007 13 797 806 10.1093/molehr/gam063 17766684

[B12] Aghajanova L Giudice LC Molecular evidence for differences in endometrium in severe versus mild endometriosis Reprod Sci 2011 18 229 251 10.1177/1933719110386241 21063030 PMC3118406

[B13] Burney RO Hamilton AE Aghajanova L Vo KC Nezhat CN Lessey BA Giudice LC MicroRNA expression profiling of eutopic secretory endometrium in women with versus without endometriosis Mol Hum Reprod 2009 15 625 631 10.1093/molehr/gap068 19692421 PMC2744474

[B14] Medicine ASfR Revised American Society for Reproductive Medicine classification of endometriosis Fertil Steril 1997 67 817 821 10.1016/S0015-0282(97)81391-X 9130884

[B15] Noyes RW Hertig AT Rock J Dating the endometrial biopsy Fertil Steril 1950 1 3 25 10.1016/j.fertnstert.2019.08.079 31623748

[B16] Ramon LA Braza-Boils A Gilabert-Estelles J Gilabert J Espana F Chirivella M Estelles A microRNAs expression in endometriosis and their relation to angiogenic factors Hum Reprod 2011 26 1082 1090 10.1093/humrep/der025 21335415

[B17] Landgraf P Rusu M Sheridan R Sewer A Iovino N Aravin A Pfeffer S Rice A Kamphorst AO Landthaler M A mammalian microRNA expression atlas based on small RNA library sequencing Cell 2007 129 1401 1414 10.1016/j.cell.2007.04.040 17604727 PMC2681231

[B18] Patterson EE Holloway AK Weng J Fojo T Kebebew E MicroRNA profiling of adrenocortical tumors reveals miR-483 as a marker of malignancy Cancer 2011 117 1630 1639 10.1002/cncr.25724 21472710 PMC3051015

[B19] Mayor-Lynn K Toloubeydokhti T Cruz AC Chegini N Expression profile of microRNAs and mRNAs in human placentas from pregnancies complicated by preeclampsia and preterm labor Reprod Sci 2011 18 46 56 10.1177/1933719110374115 21079238 PMC3343068

[B20] Ma N Wang X Qiao Y Li F Hui Y Zou C Jin J Lv G Peng Y Wang L Coexpression of an intronic microRNA and its host gene reveals a potential role for miR-483-5p as an IGF2 partner Mol Cell Endocrinol 2011 333 96 101 10.1016/j.mce.2010.11.027 21146586

[B21] Qiao Y Ma N Wang X Hui Y Li F Xiang Y Zhou J Zou C Jin J Lv G MiR-483-5p controls angiogenesis in vitro and targets serum response factor FEBS Lett 2011 585 3095 3100 10.1016/j.febslet.2011.08.039 21893058

[B22] Wang L Shi M Hou S Ding B Liu L Ji X Zhang J Deng Y MiR-483-5p suppresses the proliferation of glioma cells via directly targeting ERK1 FEBS Lett 2012 586 1312 1317 10.1016/j.febslet.2012.03.035 22465663

[B23] Bondza PK Maheux R Akoum A Insights into endometriosis-associated endometrial dysfunctions: a review Front Biosci (Elite Ed) 2009 1 415 428 19482656 10.2741/E38

[B24] Lu J Getz G Miska EA Alvarez-Saavedra E Lamb J Peck D Sweet-Cordero A Ebert BL Mak RH Ferrando AA MicroRNA expression profiles classify human cancers Nature 2005 435 834 838 10.1038/nature03702 15944708

[B25] Volinia S Calin GA Liu CG Ambs S Cimmino A Petrocca F Visone R Iorio M Roldo C Ferracin M A microRNA expression signature of human solid tumors defines cancer gene targets Proc Natl Acad Sci USA 2006 103 2257 2261 10.1073/pnas.0510565103 16461460 PMC1413718

[B26] Mitchell PS Parkin RK Kroh EM Fritz BR Wyman SK Pogosova-Agadjanyan EL Peterson A Noteboom J O'Briant KC Allen A Circulating microRNAs as stable blood-based markers for cancer detection Proc Natl Acad Sci USA 2008 105 10513 10518 10.1073/pnas.0804549105 18663219 PMC2492472

[B27] Hatziapostolou M Polytarchou C Aggelidou E Drakaki A Poultsides GA Jaeger SA Ogata H Karin M Struhl K Hadzopoulou-Cladaras M Iliopoulos D An HNF4alpha-miRNA inflammatory feedback circuit regulates hepatocellular oncogenesis Cell 2011 147 1233 1247 10.1016/j.cell.2011.10.043 22153071 PMC3251960

[B28] Ohlsson Teague EM Van der Hoek KH Van der Hoek MB Perry N Wagaarachchi P Robertson SA Print CG Hull LM MicroRNA-regulated pathways associated with endometriosis Mol Endocrinol 2009 23 265 275 19074548 10.1210/me.2008-0387 PMC5419313

