# Erratum: MicroRNAs expression profiling of eutopic proliferative endometrium in women with ovarian endometriosis

**DOI:** 10.1186/s12958-015-0040-1

**Published:** 2015-05-27

**Authors:** Piotr Laudanski, Radoslaw Charkiewicz, Mariusz Kuzmicki, Jacek Szamatowicz, Alicja Charkiewicz, Jacek Niklinski

**Affiliations:** Department of Perinatology, Medical University of Bialystok, ul. Marii Sklodowskiej-Curie 24a, 15-276 Bialystok, Poland; Department of Clinical Molecular Biology, Medical University of Bialystok, ul. Waszyngtona 13, 15-276 Bialystok, Poland; Department of Gynecology and Gynecological Oncology, Medical University of Bialystok, ul. Marii Sklodowskiej-Curie 24a, 15-276 Bialystok, Poland; Department of Pathology Medical University of Bialystok, ul. Waszyngtona 13, 15-276 Bialystok, Poland

After publication of this work [1], we noted that we inadvertently included incorrect grant numbers in the Acknowledgement section. The correct sentence should be: The study was financed by the National Science Centre within the funds of grant no. N N407 571938.
